# Design of Electronic Nose Based on MOS Gas Sensors and Its Application in Juice Identification

**DOI:** 10.3390/s25041205

**Published:** 2025-02-16

**Authors:** Yafei Zhang, Yongli Zhao, Feiyang Jiang, Rongjie Lai

**Affiliations:** School of Mechanical and Automotive Engineering, Shanghai University of Engineering Science, Shanghai 201620, China; a945993@163.com (Y.Z.); 18321260871@163.com (F.J.); lairongjie2025@163.com (R.L.)

**Keywords:** electronic nose, feature extraction, classification recognition, juice identification

## Abstract

Due to its advantages of fast response, low cost, low power consumption, and easy integration, Metal Oxide Semiconductor (MOS) gas sensor is widely used in the electronic nose system (E-nose). However, the MOS sensor has cross-sensitivity to different gases, which can impair the performance of the E-nose. Another key factor affecting the E-nose performance is the extraction method of gas features. In order to overcome the above shortcomings, an E-nose system that can modulate the operating temperature of gas sensors during the gas detection was designed in this paper, and a new gas feature extraction algorithm named Boruta-Recursive Feature Elimination (Boruta-RFE) was proposed based on the designed system. In order to verify the effectiveness of the designed system and the gas feature extraction algorithm, they were applied to the identification of different categories of apple juice. The experimental results show that more gas features can be obtained by modulating the operating temperature of the gas sensors, and the Boruta-RFE algorithm can effectively reduce the dimensionality of the original gas feature dataset, and can quickly select the key gas features, so as to effectively improve the identification accuracy of the E-nose system.

## 1. Introduction

Electronic nose (E-nose) is an artificial system that mimics the biological sense of smell. It can generate a unique fingerprint for the odor to be detected. By analyzing this fingerprint and combining with machine learning algorithms, it could quickly identify the characteristics and categories of the detected odor [1,2]. Nowadays, E-nose has attracted extensive attention and research due to its advantages of rapidity and non-destructive detection, and has been applied to many fields, such as food industry [3,4,5,6], environmental monitoring [7,8,9], cosmetics, and fragrances [10,11].

The gas sensor array, along with the associated feature extraction algorithm, constitutes the core components of E-nose technology. When exposed to gases, the gas sensor array generates a unique fingerprint resulting from the interactions of multiple sensors. The advantage of gas sensor arrays lies in their ability to provide comprehensive gas information through the collaborative action of multiple sensors, thereby enhancing the accuracy and reliability of odor recognition. Dan Wu et al. [12] successfully distinguished fruit at different stages of maturity by analyzing the aroma variations of bayberry using a sensor array (E-nose) combined with GC-MS. Emre Ordukaya and Bekir Karlik [13] employed a sensor array (E-nose) to analyze odor-based fruit juice–alcohol mixtures, using machine learning techniques for classification and Halal authentication.

Theoretically, any type of gas sensor could be employed in E-nose system. For example, Metal Oxide Semiconductor Sensor (MOS), Electrochemical Sensor (ES), Quartz Crystal Microbalance Sensor (QCMS), Surface Acoustic Wave Sensor (SAWS), Conductive Polymer Sensor (CPS), and Optical Gas Sensor (OGS). Among them, MOS sensors are widely used due to their fast response, low cost, low power consumption, and easy integration [14,15,16]. However, MOS sensors have the disadvantage of cross-sensitivity to different gases, that is the poor selectivity for gases, which in turn affects the identification accuracy of the E-nose system. One traditional way to solve this problem is to increase the number of gas sensors in the E-nose system to collect more gas information, but this inevitably increases the cost of the hardware system and the complexity of the subsequent data processing and identification algorithms.

The ability of MOS gas sensors to sense gases is based on the reaction of gas molecules with the metal oxide materials. When gas molecules are adsorbed on the surface of the metal oxide sensitive material, they react with the oxygen ions, which in turn changes the conductivity of the material. Many studies [17,18] have confirmed that there are significant differences in the surface adsorption reaction and sensitivity characteristics of MOS gas sensors at different operating temperatures. Therefore, by adjusting the operating temperature, more sensor selectivity and sensitivity characteristics could be exhibited, which is expected to obtain more gas information even in the case of a small number of sensors. Therefore, the design complexity of the E-nose system could be simplified by modulating the operating temperature.

Another important factor affecting the performance of the E-nose is the feature extraction algorithm. In practical applications, the original signal collected by the electronic nose contains a large amount of noise and redundant information, which will greatly weaken the recognition accuracy and system robustness of the electronic nose [19]. The traditional feature selection or dimensionality reduction methods, such as principal component analysis (PCA) and Least Absolute Shrinkage and Selection Operator (LASSO), can eliminate the noise and the redundant information to some extent. However, these methods perform unsatisfactorily when dealing with high-dimensional data [20,21,22]. Therefore, how to efficiently extract the most important odor features from the original data has always been a hot topic in the field of electronic nose research.

Based on the research background mentioned above, an electronic nose system based on MOS gas sensors was designed in this paper, which can adjust the operating temperature of the gas sensors in the detection process. Firstly, after collecting the response signals of target gas by using the designed electronic nose, a new feature extraction algorithm named as Boruta-RFE was proposed to reduce the dimensionality of the gas data, and the features with high contribution to the classification and recognition were evaluated and selected. Afterwards, the recognition model of the electronic nose was trained by using the selected feature dataset. Finally, in order to verify the effectiveness of the designed electronic nose system and the feature extraction algorithm, they were applied to the identification of different apple juices. The experimental results showed that the ability of the electronic nose to obtain gas information can be further improved through the modulation of the gas sensors operating temperature, and the proposed Boruta-RFE algorithm could quickly and accurately extract the key features of the detected gases, so as to effectively improve the identification accuracy of the electronic nose.

## 2. Design Methodology

### 2.1. Design of the E-Nose System

**Figure 1:**
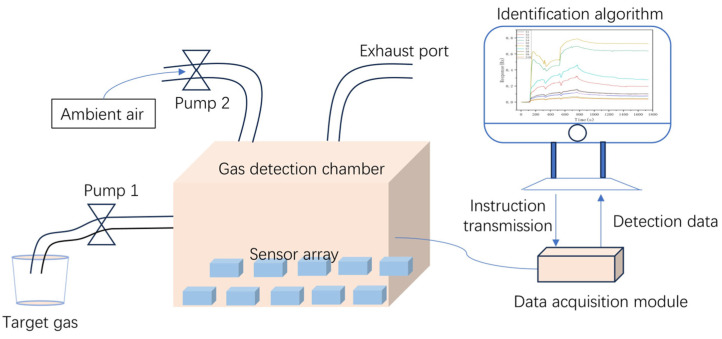
The schematic of the as designed E-nose system.

As shown in Figure 1, the E-nose system designed in this paper consists of three parts, the gas delivery module, the data acquisition module, and the identification algorithm. The gas delivery module transports target gases to the detection chamber (volume 1.88 L) via the pump 1, and clean ambient air is used to purge the detection chamber with the help of the pump 2. The data acquisition module is responsible for collecting the gas information sensed by the gas sensors. The identification algorithm completes the gas feature extraction and the classification and identification.

**Figure 2:**
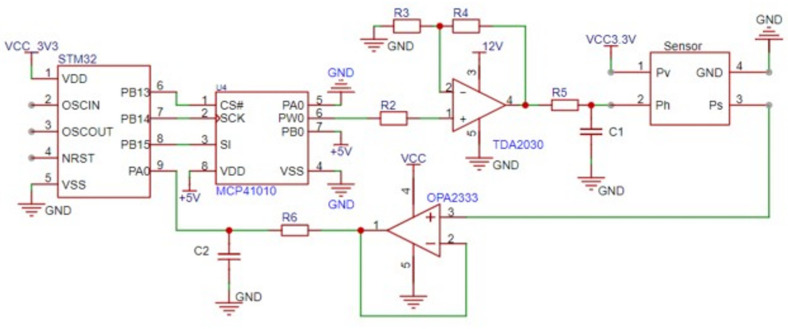
The design of the temperature modulation and signal detection circuit in the E-nose.

Ten laboratory-made MOS gas sensors were employed in the designed electronic nose system. These sensors were prepared by a thin-film process and with seven layers structure, the silicon substrate, the supporting layer (2 uµm silicon nitride and silicon oxide composite layer), the layer of micro heater (platinum wire with the power consumption about 30 mW), isolation layer (insulating material), the layer of electrodes, the protecting layer, and the sensing layer (gas sensitive material based on SnO_2_). In order to realize the gas detection of the electronic nose at different operating temperatures, a temperature modulation circuit was designed in this paper, as shown in Figure 2. This circuit used a microcontroller (STM32, Manufactured by STMicroelectronics, headquartered in Geneva, Switzerland.), a digital potentiometer (MCP41010, Manufactured by Microchip Technology Inc., headquartered in Chandler, Arizona, USA.), and a power amplifier (TDA2030, Manufactured by SGS Microelettronica (now part of STMicroelectronics), headquartered in Milan, Italy.) to regulate the voltage applied on the sensor microheater to control the operating temperature of the gas sensors. Furthermore, multiple temperature modulation channels were designed to realize independent control of the operating temperature of each sensor, and the heating voltage adjustment range is 1.0 to 2.5 V. The response signal of each sensor to the detected gas is acquired by the ADC with the detection voltage fixed on 3.3 V. Due to the impact of the ADC input impedance on the measurement accuracy of high-impedance sensors, an OPA2333-based voltage follower was employed as a buffer to isolate the ADC’s input impedance from the measurement system. The sampling frequency of the designed E-nose was set as 1 Hz.

### 2.2. Design of Feature Extraction Algorithm

**Figure 3:**
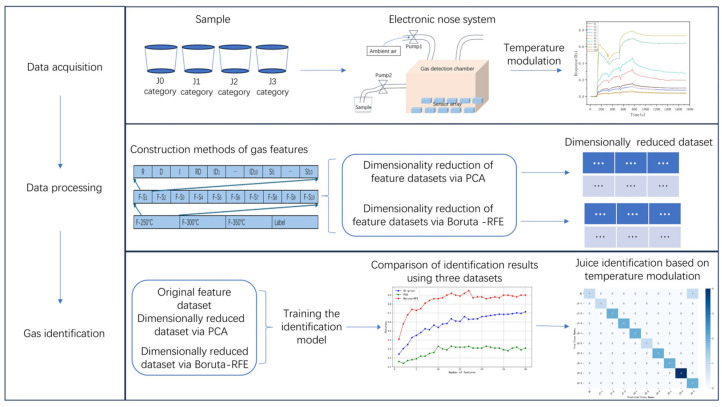
Flowchart of the juice identification approach based on the designed Boruta-RFE algorithm and the E-nose.

Figure 3 shows the whole flowchart of the electronic nose proposed in this paper, which includes three main processes: data acquisition, data processing, and gas identification. Data acquisition is a hardware system designed to obtain the response signal of each sensor to the detected gas. Data preprocessing filters the noise information from the collected response signals, which is beneficial for the feature extraction of gas samples. In order to train the identification model, the first step that needs to be done in this process is to extract as many key features that can represent the target gas as possible. However, the feature dataset composed of these extracted features usually has a higher dimension, so in order to shorten the computational time and reduce the complexity of the identification model, the dimensionality reduction algorithm is then carried out to optimize the feature dataset. Finally, in order to realize the identification of the target gas, the classification algorithm was used to perform pattern recognition on the optimized feature dataset.

### 2.3. Boruta Algorithm

In the current study, the random forest-based Boruta algorithm was applied to select the gas features that, have significant influences on the identification accuracy. The process involves generation of Shadow Features, calculating feature importance scores, conducting comparative analyses against Shadow Features, and iteratively eliminating irrelevant features based on their importance scores. The main computational procedures of the Boruta algorithm are as follows [23,24]:

Suppose the feature matrix is denoted as $X\in R^{M\times N}$, where M represents the number of samples, N is the number of features. The target variable y marks the labels of the target gas categories in the feature vector.

1. Generation of shadow features: Each column of the original feature dataset is denoted as $X_{j}$. Shadow features are generated by randomly permuting the elements within $X_{j}$, applying the permutation independently to each feature column. For each feature column, a random number generator shuffles the order of the column, resulting in a random rearrangement of each element’s position. All feature columns undergo independent permutation operations, ensuring that the shuffling of each column is independent, thereby generating the corresponding shadow features, denoted as $X_{j}^{shadow}$.

These shadow features and the original features are combined column-wise to form an extended matrix $X_{extended}$, which includes both the original feature columns and their corresponding shadow feature columns. Ultimately, the size of the matrix is M×2N, where M represents the number of samples and N denotes the number of features. Thus, the extended matrix contains each original feature column alongside its corresponding shadow feature column.

(1) $$X_{extended}=[X,X_{shadow}]$$

Since the Shadow Features are generated randomly, they have no meaningful relationship with the target variable y. Their function is to be the reference for evaluating the importance of the features, thereby helping to determine which original features are closely relevant to the target variable y.

2. Training the random forest model: A Random Forest model is trained using the extended matrix $X_{extended}$ and the target variable y.
3. Calculating feature importance scores based on the Random Forest Model: In the Random Forest model, the Gini importance is used to calculate the feature importance scores of each feature $X_{j}$ in the extended matrix $X_{extended}$.
  (2) $$I\left(X_{j}\right)\;=\frac{1}{N_{t}}\sum_{t=1}^{N_{t}}\Delta I_{s\left(t\right)}\left(X_{j}\right)$$

The $N_{t}$ denotes the number of decision trees (a parameter in the algorithm, n_estimators, with a common value of 100). $\Delta I_{s\left(t\right)}\left(x_{j}\right)$ refers to the split gain contributed by feature $X_{j}$ when it is involved in a node split within the decision tree t. This formula quantifies the average contribution of the feature across all decision trees. A higher value indicates a greater importance of the feature in the classification decision.

4. Conducting comparative analyses against shadow features: The importance of each original feature $I\left(X_{j}\right)$ is compared with the highest importance value of the shadow features, denoted as $I_{\max\_shadow}$. The comparison is performed as follows:

If $I\left(X_{j}\right)\;$≤$\;I_{\max\_shadow}$, the feature is labeled as “Irrelevant” (Rejected).

If $I\left(X_{j}\right)\;$> $I_{\max\_shadow}$, the feature is labeled as “Important” (Confirmed).

If $I\left(X_{j}\right)$ is similar to $I_{\max\_shadow}$, the importance of the feature remains inconclusive, and it is labeled as “Tentative”.

5. Iterative update: For the “Tentative” features, Boruta generates new shadow features and retrains the Random Forest model for Conducting Comparative Analyses. This process is iteratively repeated until all features are labeled as “Important” or “Irrelevant”, or the maximum number of iterations is reached.
6. If the importance of the ‘Tentative’ features has not been ascertained after reaching the predetermined maximum number of iterations, the average importance score of these features across all iterations is calculated. A threshold is then established, and if the average score exceeds this threshold, the feature is labeled as “Important”; otherwise, it is classified as “Irrelevant”.

The Boruta algorithm is capable of identifying all features in the dataset that are significantly relevant to the target variable y. The advantage of this approach is that it preserves all features that are relevant to the target variable y, rather than merely focusing on finding an optimal subset of features. This comprehensive feature selection method enhances the performance and generalization capabilities of machine learning models, rendering them more effective in handling high-dimensional and noisy data.

### 2.4. Boruta- RFE Algorithm

**Figure 4:**
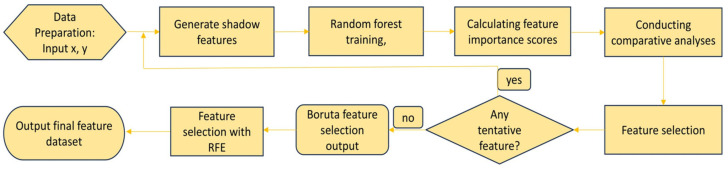
Flowchart of the feature extraction based on the Boruta-RFE algorithm.

As shown in Figure 4, the combination of Boruta and RFE facilitates a more comprehensive feature selection and dimensionality reduction:

1. Initial Screening with Boruta: Initially, Boruta is utilized for the preliminary feature selection, eliminating features with low importance in the Random Forest model. At this juncture, many features that remain significantly relevant to the target variable may persist, resulting in a feature dataset with relatively high dimensionality.
2. Further Dimensionality Reduction with RFE: Following Boruta ’s filtering of the feature dataset, the RFE method is applied. RFE recursively eliminates the least important features to further reduce the feature count. The number of iterations corresponds to the difference between the original feature count and the number of features retained at the conclusion.

## 3. Experiment

At present, electronic nose has been widely used in the food industry. For example, De-La-Cruz et al. [25] successfully distinguished Pisco (a Peru beverage) from different origins using an electronic nose and demonstrated that using augmented data to train the model showed better performance than using original data. Rasekh et al. [26] recognized several fruit juices by combining electronic noses with artificial neural networks. And Ma et al. [27] predicted the quality changes of fresh watermelon juice under different storage conditions also using an electronic nose. Therefore, in order to verify the effectiveness of the electronic nose system designed in this paper, we applied it to the identification of different types of fruit juices.

### 3.1. Materials

Four categories of apple juice were prepared in the current study: freshly squeezed apple juice, freshly squeezed apple juice mixed with purified water, and two different brands of apple juice respectively. The freshly squeezed juice was made from red Fuji apples (Luochuan CityShanxi province, China), and the two juices on sale were Huiyuan brand concentrated apple juice and Huierkang brand red apple juice. Fresh apples were firstly peeled and then squeezed using a centrifugal juicer. Freshly squeezed apple juice was kept in several sealed bottles and then mixed with other liquids (purified water or one apple juice on sale) with different proportions. Furthermore, three sub-categories of samples containing different volume ratios (10%, 20%, and 30%, respectively) were prepared for obtaining different apple juices. In addition, the pure freshly squeezed apple juice was set up as one category. Therefore, a total of ten categories of samples were detected using the designed E-nose (Each category consisted of ten samples.). The details of all samples are listed in Table 1.

As shown in Table 2, ten sensors were employed in this study to ensure that each sensor would respond to the experimental samples. Ten self-made metal oxide semiconductor gas sensors were selected to form the sensor array of the electronic nose. These sensors feature a multilayer structure and were fabricated using thin-film technology.

### 3.2. Experimental Procedure

The designed electronic nose system was used to detect the experimental samples in the laboratory environment with temperature of 20 ± 2 °C and humidity of 65 ± 5%RH, and the detailed experimental steps are as follows:

- After the gas sensors were preheated, clean air is introduced into the sensor chamber to remove impurity gases, waiting for all the sensors to reach a stable baseline state.
- The gas in the headspace of the sealed bottle is pumped by an air pump and delivered into the detection chamber for 600 s. During the detection process, the operating temperature of the sensors is changed from 250 to 350 °C.
- After the detection is complete, clean air is introduced again to clean the detection chamber until the sensor response curve returns to baseline.
- Repeat the above steps to complete the detection of all samples.

## 4. Results and Discussion

### 4.1. Response of the E-Nose

In the actual application environment, the response curve of the sensor to the target gas fluctuates due to the influence of temperature and humidity [28]. In addition, as the sensors are composed of different materials, there are some differences in their response values [29,30]. In order to facilitate the analysis of the response data, it was preprocessed using the follow formula:

(3) $$R_{s}=\frac{R_{0}-R_{t}}{R_{0}}$$

where $R_{s}$ is the response sensitivity, $R_{t}$ is the real-time resistance value of the sensor, and $R_{0}$ is the resistance value of the sensor at steady state. By converting the original data into dimensionless standardized values, the computational complexity caused by the difference magnitude of the data can be reduced.

**Figure 5:**
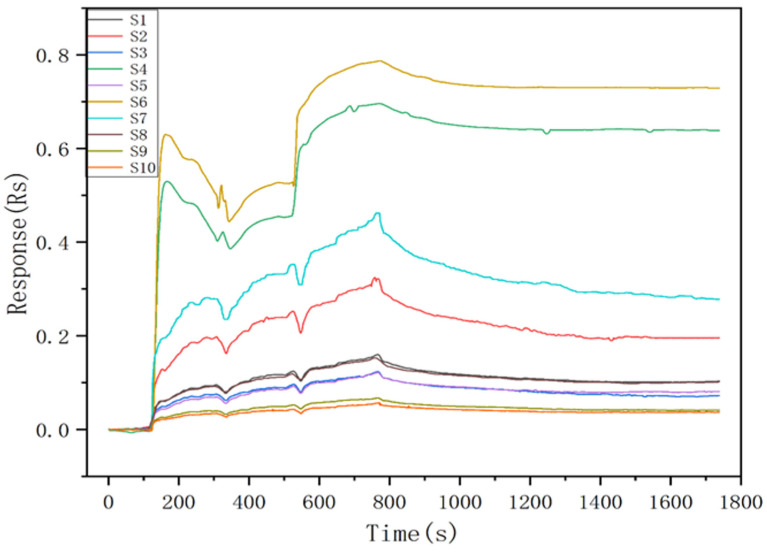
The response curves of the E-nose after the normalization using Formula (3).

Figure 5 shows the response curves of the electronic nose to the experimental sample gas. The experimental gas begins to be delivered into the detection chamber at the time of 100 s. It is can be seen all the sensors respond immediately and rise rapidly when the experimental gas is introduced into the detection chamber. and in each stage, the maximum value is reached after approximately 200 s. After the delivery of the experimental gas was stopped and fresh air was subsequently introduced to clean the detection chamber, the response curves also showed rapid decreases until returned to their initial steady state. This indicates that the designed E-nose has a good response to the target gas. In Figure 5, the time range from 100 s to 700 s, totaling 600 s, is divided into three intervals: [100, 300], [300, 500], and [500, 700]. The operating temperatures for these intervals are 250 °C, 300 °C, and 350 °C, respectively, with each stage lasting 200 s.

### 4.2. Construction of Gas Features

**Figure 6:**
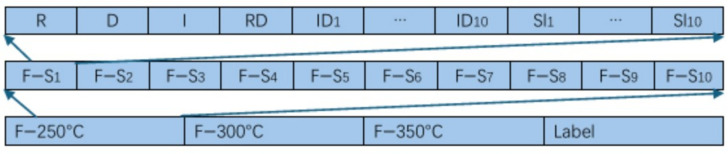
Schematic of the feature vector organization in the feature dataset.

Table 3 lists the features extracted from the response curve of each sensor. A total of 24 features are considered in the current study, and they are sorted according to one-dimensional vectors to construct the feature dataset, as shown in Figure 6. The designed electronic nose contains ten gas sensors, therefore the total count of the extracted features for each sample at each operating temperature is equal to 240 (24 × 10 = 240). And because three operating temperatures (250, 300, and 350 °C) were experimented in the current study, the total number of features extracted from each sample is equal to 720 (240 × 3 = 720).

**Figure 7:**
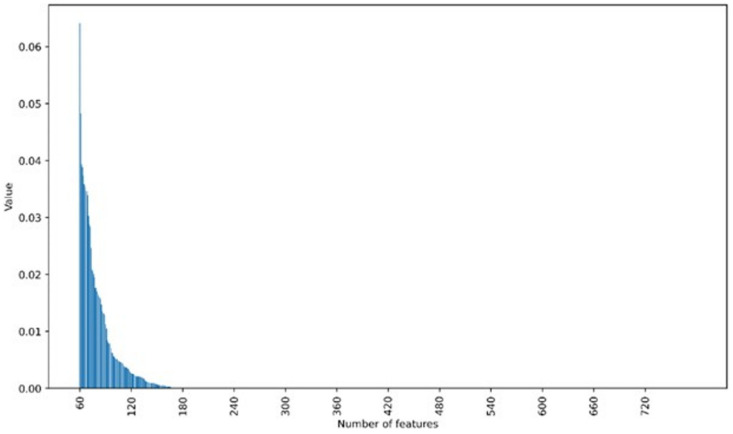
The importance values of the extracted gas features based on Boruta algorithm.

In order to explore the importance of the extracted features to the classification and recognition, the Boruta algorithm was used to analyze the original feature dataset. As can be seen from Figure 7, only 30 of the 720 features have a weight value higher than 0.01. These features with high weights play a key role in the classification and recognition. Therefore, the selection of features through an appropriate algorithm by retaining the features with higher weights and eliminating the features with smaller weights will help to simplify the recognition model. Interestingly, of these top 30 features, 16 are curve slope and interval difference features. These two types of features express the dynamic change process of the response curve, which indicates that the transient change of the response curve is the key factor for the identification of gases.

### 4.3. Dimensionality Reduction of Feature Datasets

#### 4.3.1. PCA Method

**Figure 8:**
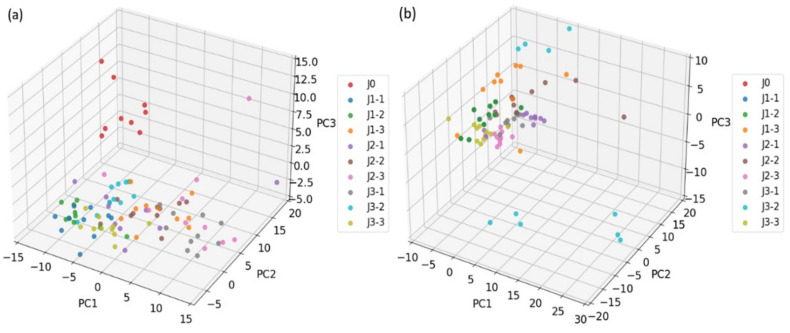

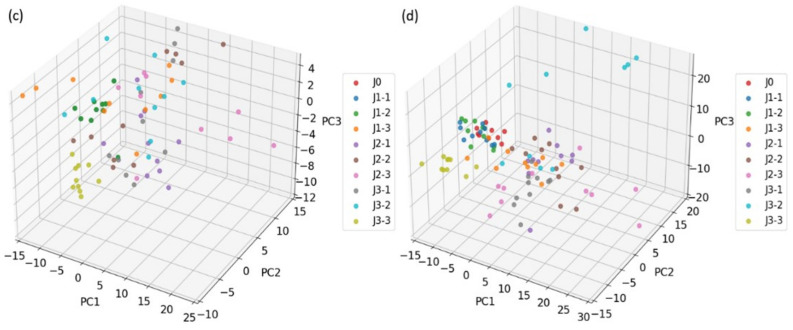
Distribution of the samples detected on different operating temperatures after the dimensionality reduction using PCA: (**a**) 250 °C, (**b**) 300 °C, (**c**) 350 °C, and (**d**) on temperature modulation.

The purpose of the dimensionality reduction on the feature datasets is to reduce the computational complexity by simplifying the data structure while retaining the key information of the original dataset as much as possible. In order to compare the effectiveness of our proposed Boruta-RFE algorithm on the dimensionality reduction and the selection of gas feature datasets, we firstly use the traditional PCA method to analyze the original feature datasets. The results show that the first three principal components of PCA can explain 81% of the variance. As can be seen from the 3D scatter plots (see Figure 8) of the PCA analysis, there are obvious overlaps between the different categories of samples. This result is consistent with the findings of Haowu et al. [31,32], which suggested that while principal components can explain most of the variance of the feature dataset, they may still not be effective in distinguishing different sample categories.

#### 4.3.2. Boruta-RFE Method

**Figure 9:**
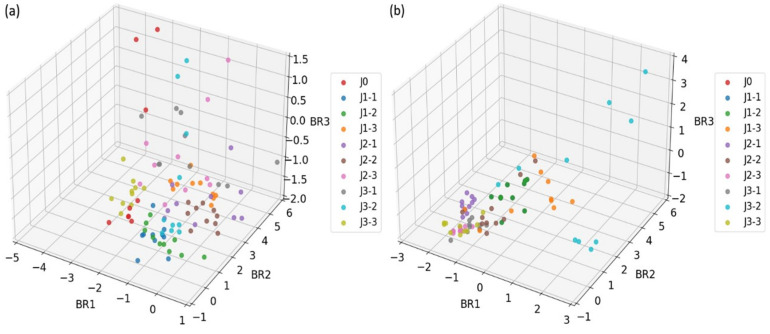

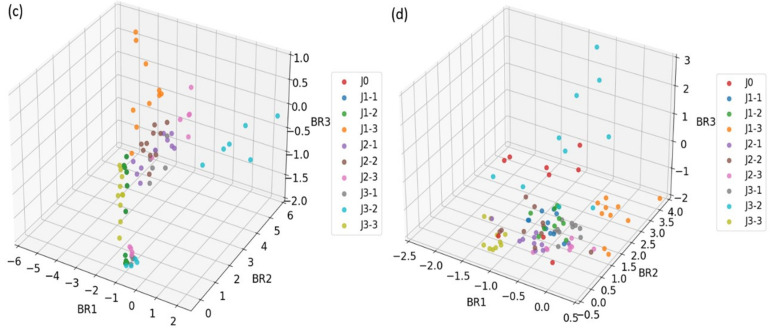
Distribution of the samples detected on different operating temperatures after the dimensionality reduction using Boruta-RFE: (**a**) 250 °C, (**b**) 300 °C, (**c**) 350 °C, and (**d**) on temperature modulation.

Figure 9 shows the distribution of different categories after the dimensionality reduction using as proposed Boruta-RFE algorithm. Compared with the PCA method, although there is still overlap between different categories, the sample distribution of the same category is more compact, and the intra-category aggregation is significantly improved, which indicates that Boruta-RFE has advantages in reducing feature redundancy and selecting the key features from the original feature dataset. Furthermore, from the 3D scatter plot after the Boruta-RFE dimensionality reduction on the feature dataset, it can be intuitively observed that the difference of category classification between the detection under a single operating temperature and under the modulation operating temperature. At a single operating temperature, the Boruta-RFE has shown good classification efficacy, although several of samples (e.g., categories J1 and J2, J2 and J3) are still loosely distributed. As more gas information can be obtained under temperature modulation conditions, which increases the expressive ability of gas feature dataset. Therefore, this not only significantly improves the tightness of the sample distribution within the same category, but also strengthens the dispersion effect between different categories. Especially for those categories with small differences in composition (e.g., classes J1-2 and J1-3, J2-2 and J2-3), temperature modulation can be used to distinguish these categories more clearly.

### 4.4. Identification Result

In this study, the XGBoost(Run in Jupyter notebook 7.1.2) algorithm was used to train the identification model. GridSearchCV was used for tri-fold cross-validation, and the identification accuracy was used to evaluate the optimal parameter combination. After finding the best parameter combination, five-fold cross-validation was then applied to obtain the accuracy for each feature dimension. The model parameters were set as follows: the learning rate is set to 0.2, the maximum depth of the tree is 5, the model contains 100 trees, and 80% of the samples were selected randomly and were used to train the model.

**Figure 10:**
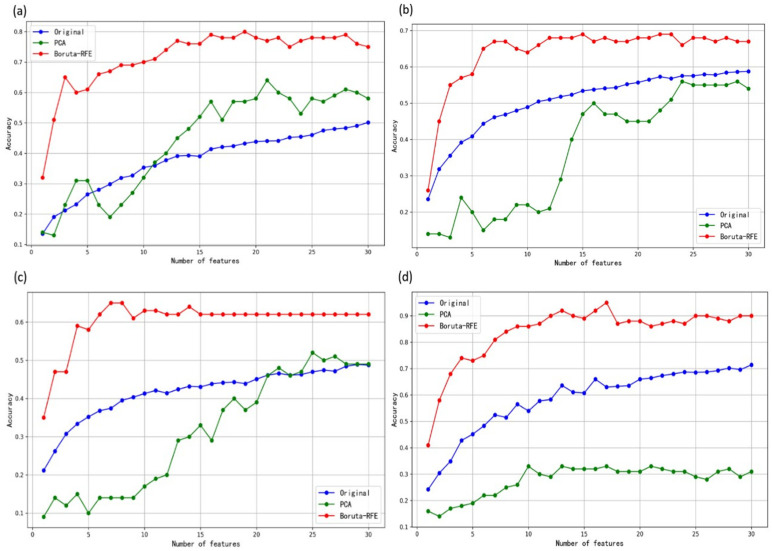
Identification accuracies of the E-nose based on the gas feature dataset processed by different dimensionality reduction methods: (**a**) 250 °C, (**b**) 300 °C, (**c**) 350 °C, and (**d**) temperature modulation.

As can be seen from Figure 10, for the PCA method, the identification accuracy increases with the increase of the number of features in the temperature modulation mode (from 19% for 5 features to 30% for 30 features), but the identification accuracy does not improve after the use of higher-dimensional features. This is because the PCA method mainly selects principal components based on the variance of the feature dataset, and does not fully consider the differences information between the different categories. More features (720) could be extracted when the sample was detected with the operating temperature changing, which may contain more redundant features and thereby weaken the extraction of key features by using PCA analysis. This phenomenon matches the study of Cunningham et al. [33], which showed that redundant features can significantly affect classification performance in high-dimensional datasets.

Figure 10 also shows the identification accuracy of the feature dataset based on the original feature dataset and the feature dataset analyzed by the Boruta-RFE algorithm (“Original” refers to the feature dataset that has not undergone any dimensionality reduction. Specifically, the process involves randomly selecting a subset of dimensions from the feature dataset, performing 30 independent modeling runs, and then averaging the results from all the models.). It can be seen that the Boruta-RFE algorithm shows the best results. This indicates that it has a good ability to extract key features from the original dataset. At a low number of features (less than 10 features), the identification accuracy based on the Boruta-RFE dataset increases rapidly and achieves the best accuracy with 10–15 features. With more addition features, the improvement in identification accuracy is not obvious. This confirms that the Boruta-RFE algorithm can extract the features that are most useful for identification task, without adding more redundant features that affect the E-nose performance.

The 12-feature model achieved an accuracy of 90%, with a confidence interval of [68.3%, 98.77%], while the 17-feature model achieved an accuracy of 95%, with a confidence interval of [75.13%, 99.87%]. Although the 17-feature model shows a slight improvement in accuracy, this improvement is not statistically significant when compared to the 12-feature model. While smaller models, such as the 12-feature model, tend to perform better in terms of stability and robustness, the 17-feature model was ultimately selected for training due to its higher accuracy, as accuracy is the primary evaluation metric. Additionally, increasing the number of experimental samples would enhance the statistical confidence of the results.

**Figure 11:**
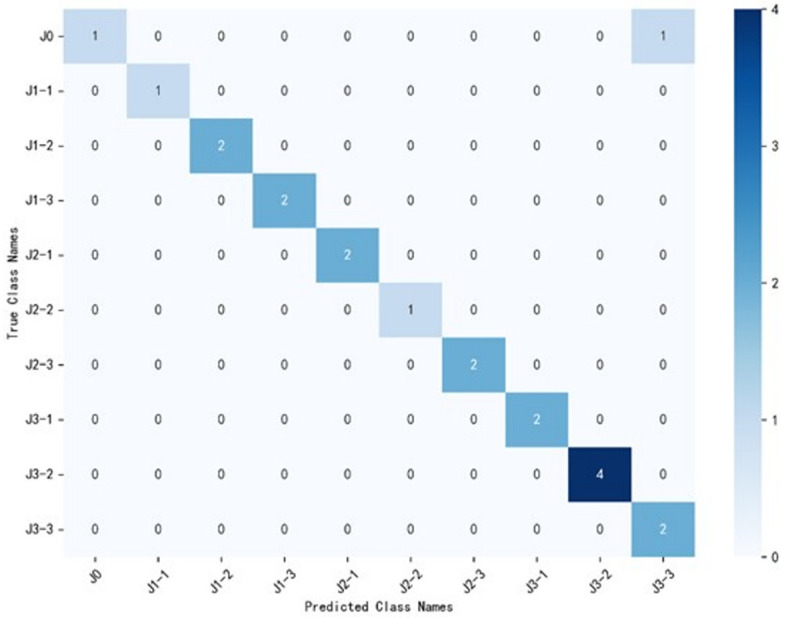
Confusion matrix of the juice identification based on temperature modulation dataset.

Based on the temperature modulation dataset, 17 features were ultimately selected to train the classification model, which obtained the identification accuracy of 95%. Figure 11 shows the confusion matrix of the identification process. The big numbers along the diagonal indicate that the model exhibits excellent identification performance. Notably, only one sample from the J3-3 category was misclassified as J0. This result demonstrates that temperature modulation could provide much more information that in turn enhance the discrimination between the categories with very subtle small differences.

### 4.5. Effect of the Operating Temperature of Gas Sensors

**Figure 12:**
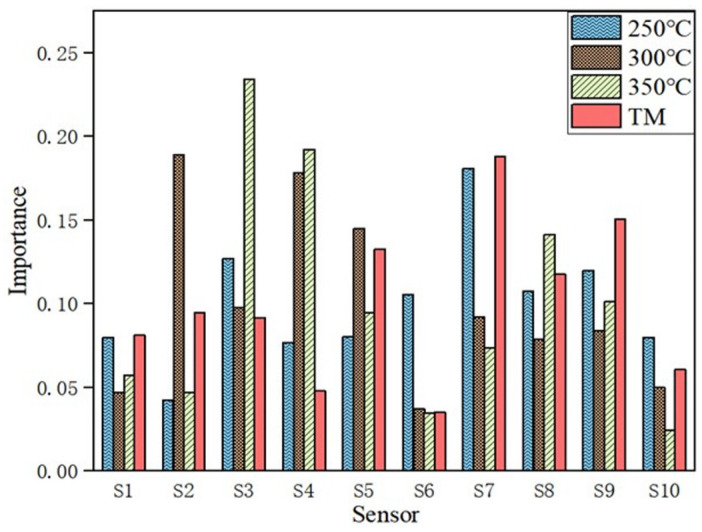
Feature weight values of the sensor contributions in the four datasets.

Figure 12 shows the sum of the feature importance of all the gas sensors in the four datasets (“Importance” refers to the sum of each sensor’s contribution to all the features.). It can be observed there is a significant difference in the response contribution between the sensors when they worked at different operating temperatures. Sensor S3 has the highest contribution (0.2339) at 350 °C, indicating that it has the best sensitivity under this temperature, S2 exhibits its peak contribution of 0.1889 at 300 °C, confirming that it performs more prominently in the medium temperature range, and S7 and S9 have higher contributions under temperature modulation (TM) conditions (0.1879 and 0.1504), respectively, showing that the modulation of the operating temperature could effectively arouse their response to target gases. In addition, the contribution of sensor S6 is relatively low at all temperatures. This result indicates that its response sensitivity to the gas of the current experimental samples is weaker than that of other sensors. In summary, each sensor has different sensitivities at different operating temperature and has its own optimal operating temperature range. Therefore, the sensors should be adapted according to their operating temperature characteristic so as to improve the performance of the E-nose system.

## 5. Conclusions

In this paper, an E-nose system based on MOS gas sensor was successfully designed, which can modulate the operating temperature of the gas sensor by changing the voltage of the microheater during the gas detection. Based on the designed E-nose system, a new gas feature extraction method Boruta-RFE is proposed to analyze the gas characteristic dataset. The main results are: (1) modulating the operating temperature of the gas sensors can obtain more gas information than that of any fixed operating temperature. (2) Compared with the traditional PCA method, the proposed Boruta-RFE algorithm can effectively select the key gas features with high contribution to the classification, so as to effectively improve the identification accuracy of the E-nose.

## Figures and Tables

**Table 1:** The composition of the experimental samples.

Category No.	Freshly Squeezed Apple Juice (Vol-%)	Purified Water (Vol-%)	Huiyuan Apple Juice (Vol-%)	Huierkang Apple Juice (Vol-%)
J0	100	0	0	0
J1-1	90	10	0	0
J1-2	80	20	0	0
J1-3	70	30	0	0
J2-1	90	0	10	0
J2-2	80	0	20	0
J2-3	70	0	30	0
J3-1	90	0	0	10
J3-2	80	0	0	20
J3-3	70	0	0	30

**Table 2:** Characteristics of the employed sensors in E-nose.

Sensor No.	Materials	Main Detected Gas
S1	Pt/SnO_2_	Ethanol, Acetaldehyde, Carbon monoxide
S2	Pt/SnO_2_	VOCs, Ethanol, Acetone, Hydrogen, Methane
S3	Pd/SnO_2_	Carbon monoxide, Ethanol
S4	Pd/SnO_2_	Methane, Hydrogen sulfide, Ethanol
S5	ZnO/SnO_2_	VOCs
S6	ZnO/SnO_2_	Aldehydes, Ketones
S7	ZnO/SnO_2_	Aldehydes, Ketones
S8	NiO/SnO_2_	Ethanol, Ammonia
S9	SnO_2_/MWCNT	Hydrogen sulfide, Acetone, Ethanol
S10	SnO_2_/MWCNT	Acetone, Hydrogen sulfide, Ethanol

**Table 3:** The construction methods of gas features.

Feature Label	Feature Name	Number of Features	Function
R	Maximum response	1	$R_{max}$
D	Absolute difference	1	$R_{max}$−$R_{min}$
I	Integral value	1	$\int_{0}^{200}R_{s}\left(t\right)dt$
RD	Relative difference	1	$\frac{R_{max}}{R_{min}}$
ID1–ID10	(Interval difference)	10	$R_{i+20}-R_{i}$
Sl1–Sl10	Curve slope	10	$\frac{ID_{i}}{20}$

## Data Availability

Data are contained within the article.

## References

[1] Villarrubia G., De Paz J.F., Pelki D., de la Prieta F., Omatu S. (2017). Virtual organization with fusion knowledge in odor classification. Neurocomputing.

[2] Yan J., Guo X., Duan S., Jia P., Wang L., Peng C., Zhang S. (2017). Electronic nose feature extraction methods: A review. Sensors.

[3] Wen J., Zhao Y., Rong Q., Yang Z., Yin J., Peng Z. (2022). Rapid odor recognition based on reliefF algorithm using electronic nose and its application in fruit identification and classification. Sensors.

[4] Qi P.-F., Meng Q.-H., Zhou Y., Jing Y.-Q., Zeng M. A portable E-nose system for classification of Chinese liquor. Proceedings of the 2015 IEEE Sensors.

[5] Lu L., Hu Z., Hu X., Li D., Tian S. (2022). Electronic tongue and electronic nose for food quality and safety. Food Res. Int..

[6] Baldwin E.A., Bai J., Plotto A., Dea S. (2011). Electronic noses and tongues: Applications for the food and pharmaceutical industries. Sensors.

[7] Yin J., Zhao Y., Peng Z., Ba F., Peng P., Liu X., Zhang Y. (2023). Rapid Identification Method for CH_4_/CO/CH_4_-CO Gas Mixtures Based on Electronic Nose. Sensors.

[8] Domènech-Gil G., Duc N.T., Wikner J.J., Eriksson J., Påledal S.N., Puglisi D., Bastviken D. (2023). Electronic Nose for Improved Environmental Methane Monitoring. Environ. Sci. Technol..

[9] Capelli L., Sironi S., Rosso R.D. (2014). Electronic noses for environmental monitoring applications. Sensors.

[10] Rico F., Mazabel A., Egurrola G., Pulido J., Barrios N., Marquez R., García J. (2023). Meta-Analysis and Analytical Methods in Cosmetics Formulation: A Review. Cosmetics.

[11] Suslick B.A., Feng L., Suslick K.S. (2010). Discrimination of complex mixtures by a colorimetric sensor array: Coffee aromas. Anal. Chem..

[12] Ordukaya E., Karlik B. (2016). Fruit juice–alcohol mixture analysis using machine learning and electronic nose. IEEJ Trans. Electr. Electron. Eng..

[13] Wu D., Cheng H., Chen J., Ye X., Liu Y. (2019). Characteristics changes of Chinese bayberry (*Myrica rubra*) during different growth stages. J. Food Sci. Technol..

[14] Cheng L., Meng Q.H., Lilienthal A.J., Qi P.F. (2021). Development of compact electronic noses: A review. Meas. Sci. Technol..

[15] Luan S., Hu J., Ma M., Tian J., Liu D., Wang J., Wang J. (2023). The enhanced sensing properties of MOS-based resistive gas sensors by Au functionalization: A review. Dalton Trans..

[16] Wawrzyniak J. (2023). Advancements in improving selectivity of metal oxide semiconductor gas sensors opening new perspectives for their application in food industry. Sensors.

[17] Zhang J. (2021). Effect of Co doping on chemosorbed oxygen accumulation and gas response of SnO_2_ under dynamic program cooling. Sens. Actuators B Chem..

[18] Drix D., Dennler N., Schmuker M. Rapid recognition of olfactory scenes with a portable MOx sensor system using hotplate modulation. Proceedings of the 2022 IEEE International Symposium on Olfaction and Electronic Nose (ISOEN).

[19] Peng Z., Zhao Y., Yin J., Peng P., Ba F., Liu X., Zhang Y. (2023). A Comprehensive Evaluation Model for Optimizing the Sensor Array of Electronic Nose. Appl. Sci..

[20] Fan J., Li R. (2006). Statistical challenges with high dimensionality: Feature selection in knowledge discovery. arXiv.

[21] Zou H., Hastie T., Tibshirani R. (2006). Sparse principal component analysis. J. Comput. Graph. Stat..

[22] Johnstone I.M., Lu A.Y. (2009). On consistency and sparsity for principal components analysis in high dimensions. J. Am. Stat. Assoc..

[23] Kursa M.B., Jankowski A., Rudnicki W.R. (2010). Boruta–a system for feature selection. Fundam. Inform..

[24] Kursa M.B., Rudnicki W.R. (2010). Feature selection with the Boruta package. J. Stat. Softw..

[25] De-La-Cruz C., Trevejo-Pinedo J., Bravo F., Visurraga K., Peña-Echevarría J., Pinedo A., Sun-Kou M.R. (2023). Application of machine learning algorithms to classify Peruvian pisco varieties using an electronic nose. Sensors.

[26] Rasekh M., Karami H. (2021). Application of electronic nose with chemometrics methods to the detection of juices fraud. J. Food Process. Preserv..

[27] Ma T., Wang J., Wang H., Lan T., Liu R., Gao T., Sun X. (2020). Is overnight fresh juice drinkable? The shelf life prediction of non-industrial fresh watermelon juice based on the nutritional quality, microbial safety quality, and sensory quality. Food Nutr. Res..

[28] Tong Y., Zhao B., Zhao Y., Yang T., Yang F., Hu Q., Zhao C. (2015). Novel anode-supported tubular solid-oxide electrolytic cell for direct NO decomposition in N_2_ environment. Int. J. Electrochem. Sci..

[29] Zhao Y.L., Zhao C.H., Huang J., Zhao B. (2014). LaMnO_3_–Ni_0.75_Mn_2.25_O_4_ supported bilayer NTC thermistors. J. Am. Ceram. Soc..

[30] Zhao Y., Wang Y., Peyraut F., Planche M.P., Ilavsky J., Liao H., Allimant A. (2020). Parametric analysis and modeling for the porosity prediction in suspension plasma-sprayed coatings. J. Therm. Spray Technol..

[31] Wu H., Yue T., Yuan Y. (2018). Authenticity tracing of apples according to variety and geographical origin based on electronic nose and electronic tongue. Food Anal. Methods.

[32] Wu H., Wang J., Yue T., Yuan Y. (2017). Variety-based discrimination of apple juices by an electronic nose and gas chromatography–mass spectrometry. Int. J. Food Sci. Technol..

[33] Cunningham P., Lovell B.C. (2008). Dimension reduction. Machine Learning Techniques for Multimedia: Case Studies on Organization and Retrieval.

